# Insight into the Gel Properties of Antarctic Krill and Pacific White Shrimp Surimi Gels and the Feasibility of Polysaccharides as Texture Enhancers of Antarctic Krill Surimi Gels

**DOI:** 10.3390/foods11162517

**Published:** 2022-08-20

**Authors:** Shuang Li, Songyi Lin, Pengfei Jiang, Zhijie Bao, Sibo Li, Na Sun

**Affiliations:** 1 National Engineering Research Center of Seafood, School of Food Science and Technology, Dalian Polytechnic University, Dalian 116034, China; 2 Collaborative Innovation Center of Seafood Deep Processing, Dalian Polytechnic University, Dalian 116034, China

**Keywords:** Antarctic krill, shrimp surimi, gelation, cross-linkage, polysaccharide

## Abstract

Antarctic krill is a potential and attractive resource for consumption. However, most Antarctic krill meat is used to produce primary products with low commercial value, with few highly processed products. This study aimed to evaluate and improve the gelling properties of Antarctic krill surimi, with Pacific white shrimp surimi as control. Compared with Pacific white shrimp surimi, the lower β-sheet content and protein aggregation degree had a severe impact on the formation of the gel network of Antarctic krill surimi, which resulted in weaker breaking force, gel strength, and viscoelasticity (*p* < 0.05). Moreover, water retention capacity and molecular forces had a positive effect on the stability of the gel matrix of shrimp surimi. Thus, the high α-helix/β-sheet ratio, weak intermolecular interactions, and low level of protein network cross-linkage were the main reasons for the poor quality of Antarctic krill surimi. On this basis, the effects of six polysaccharides on the texture properties of Antarctic krill surimi were studied. Chitosan, konjac glucomannan, sodium carboxyl methyl cellulose, and waxy maize starch resulted in no significant improvement in the texture properties of Antarctic krill surimi (*p* > 0.05). However, the addition of ι-carrageenan (2%) or κ-carrageenan (1~2%) is an effective way to improve the texture properties of Antarctic krill surimi (*p* < 0.05). These findings will contribute to the development of reconstituted Antarctic krill surimi products with high nutritional quality and the promotion of deep-processing products of Antarctic krill meat.

## 1. Introduction

Antarctic krill is one of the most abundant biomass resources on earth, with an estimated biomass of about 500 million metric tons [1]. From a nutritional point of view, Antarctic krill contains 11.9–15.4% protein, which contains all the amino acids the human body needs. The amount of each essential amino acid is sufficient to meet the FAO/WHO requirements for adults or infants [2]. The total essential amino acid content of Antarctic krill was 515.6 mg/g protein, which exceeded 277 (292.6) mg/g protein for the essential amino acid requirements of adults (infants). In addition, krill total fatty acids are composed of approximately 50% of polyunsaturated fatty acids (PUFAs) [3]. Therefore, Antarctic krill is a more attractive resource with more potential for consumption due to its extreme abundance and high nutritional value [4]. However, relatively rare information is available on relevant studies of heat-induced Antarctic krill surimi gel.

Shrimp surimi products, as an essential part of processed aquatic food, are widely welcomed by consumers due to their convenient consumption and high nutrition. In the market, Pacific white shrimp are often processed into various types of shrimp meat products (shrimp balls, shrimp sticks, etc.). Compared with Pacific white shrimp, Antarctic krill not only has outstanding nutritional value [2,3], but also has extremely active endogenous enzymes, which can make Antarctic krill autolyze rapidly after death [5]. This characteristic of Antarctic krill seriously affects the functional properties of its own proteins [6]. Shrimp surimi gel is a typical protein gel whose gel property depends on the shrimp proteins involved [7,8,9]. Studies have reported that myofibrillar proteins can form a three-dimensional gel matrix with viscoelastic properties [8]. At low temperature, myosin (the main component of myofibrillar proteins) dissolves and unfolds in salt, exposing hydrophobic sites. During heat-induced processes, myosin forms a rigid network due to intermolecular association and aggregation [7]. In recent years, there have been some studies on the preparation of shrimp surimi products using Pacific white shrimp as raw materials [9,10,11]. However, most Antarctic krill is used to produce animal feed, frozen krill meat, and other primary products (dried Antarctic krill, Antarctic krill paste, etc.) with low commercial value, and with few highly processed products [12]; furthermore, there are few studies on the gel quality of Antarctic krill surimi [3].

Possible solutions to improving the gel properties of Antarctic krill surimi include the application of proteolytic inhibitors or the addition of other substances (polysaccharides, salts, enzyme preparations, etc.) [3,5]. At present, polysaccharide–protein composite gel has been widely used in surimi processing. It has been reported that the meat products exhibited better gel properties with the addition of sulfate polysaccharide, modified starch, konjac glucomannan, κ-carrageenan, β-glucan, etc. [13]. Polysaccharides would improve the gel properties of surimi by providing additional bonding [14]. If this goal were to be attained, Antarctic krill could be used as the main raw material in developing recombinant Antarctic krill surimi products with high nutritional quality.

Up to now, there have been no studies on the gelling properties of heat-induced Antarctic krill surimi gel and the measures to improve its gelation quality. This study aimed to evaluate the macroscopic quality and gelling properties of Antarctic krill surimi with Pacific white shrimp surimi as the control group. With this aim, the possibility of improving the texture characteristics of Antarctic krill surimi by adding polysaccharides was explored. According to current research, the six selected polysaccharides (chitosan, konjac glucomannan, sodium carboxyl methyl cellulose, waxy maize starch, ι-carrageenan, and κ-carrageenan) represent polysaccharides from different sources and cover different ionic types (such as cationic, neutral, and anionic polysaccharides). These findings will clarify the gel properties of heat-induced Antarctic krill surimi gel, and provide a reference point for the in-depth development of Antarctic krill surimi with high nutritional quality.

## 2. Materials and Methods

### 2.1. Materials

Frozen Antarctic krill was purchased from Dalian Haiyue Shangpin International Trade Co., Ltd. (Liaoning, China). Antarctic krill was immediately shelled and washed, and cold chain transported. Frozen Pacific white shrimp was obtained from Dalian Xinzhilian Market (Liaoning, China). Antarctic krill and Pacific white shrimp are 4–5 cm and 16–20 cm in length, respectively. Both krill and shrimp were frozen at −20 °C until use but not longer than 4 months. Before the experiments, frozen samples were thawed at 4 °C for 8 h. Chitosan (CS) was purchased from Shandong Aokang Biotechnology Co., Ltd. (Jinan, China). Konjac glucomannan (KG) and waxy maize starch (WMS) were purchased from Shanghai Beilian Biotechnology Co., Ltd. (Shanghai, China). Sodium carboxyl methyl cellulose (CMC-Na) was purchased from Henan Wan Bang Industrial Co. Ltd. (Zhengzhou, China). ι-Carrageenan (ιCG) and κ-carrageenan (κCG) were purchased from Shanghai Beilian Biotechnology Co., Ltd. (Shanghai, China). All other chemical reagents (NaCl, β-Mercaptoethanol, urea, sodium dodecyl sulfate, glycerin, etc.) are of analytical grade.

### 2.2. Preparation of Shrimp Surimi Gels

Both krill and shrimp were thawed at 4 °C for 8 h before being decapitated, peeled, and chopped. The shrimp meat (approximately 300 g) was washed three times with cold water (4 °C) and mixed with 3.0% salt (1800 rpm, 2 min, < 10 °C) in a cutter. The water content of shrimp surimi was adjusted to 80% with cold water (4 °C), and mincing was performed for another 3 min. Finally, the shrimp surimi was inserted into plastic tubing with a diameter of 30 mm. A quarter of the shrimp surimi sols were kept in a 4 °C refrigerator for subsequent experiments. Three quarters of the shrimp surimi samples were heated at 40 °C for 30 min and at 90 °C for 20 min in a water bath, and then immediately placed in ice water and maintained at 4 °C for 24 h [15].

The thawed Antarctic krill meat (approximately 300 g) was prepared in the same way as that of Pacific white shrimp. Antarctic krill meat was mixed with 3.0% salt and polysaccharides were added to obtain final polysaccharide levels of 0.5, 1.0, 1.5, and 2.0% (based on shrimp surimi content). The water content of shrimp surimi was adjusted to 80%, and mincing was performed for another 3 min. Finally, the shrimp surimi was inserted into plastic tubing and prepared in the same way as that of Pacific white shrimp.

### 2.3. Texture Profile Analysis (TPA)

Gel texture analysis was conducted using a TA-XT Plus Texture Analyzer (Stable Micro Systems, Godalming, UK). The shrimp surimi gels were cut into sections (20 mm thick) and tested with a P36R cylinder (diameter: 36 mm). Then, the TPA analysis was performed with a pre-test speed (5 mm/s), a test speed (2 mm/s), a post-test speed (2 mm/s), a 60% compression ratio, and a 5 g trigger force.

### 2.4. Gel Strength

A TA-XT Plus Texture Analyzer (Stable Micro System Co., Godalming, UK) with a cylindrical probe (P/0.5, diameter: 12.7 mm) was used to determine the breaking force and deformation of the cylindrical gels with 2 cm height. Firstly, shrimp surimi gels were stored at room temperature for 2 h, and then the determination mode was set as gel strength, the testing speed was 1 mm/s, and the strain was 60%. The trigger force was 5 g, and the gel strength was equal to the breaking distance multiplied by the breaking strength.

### 2.5. Rheological Tests

The rheological properties of shrimp surimi sols were determined using a rheometer (Discovery DHR-1, New Castle, TA, USA). The specific operational conditions of 1 mm gap, 40 mm parallel plate geometry, 1 Hz, 25 °C, and 0.01 to 100% strain were used to monitor the linear viscoelastic region. The specific operational conditions for frequency sweep were as follows: 40 mm parallel plate geometry, 1 mm gap, 25 °C, 1.0% strain, and 0.1 to 10 Hz. The frequency variation of viscoelastic modulus was measured. 

Temperature sweep measurements (20 to 80 °C, 1 °C/ min) were carried out on the unheated sols. The specific operational conditions of 1 Hz, and 1% strain within the linear viscoelastic region were used to monitor storage modulus (G′), loss modulus (G”) and the loss factor during heating.

### 2.6. Water-Holding Capacity

Aliquots of approximately 5 g shrimp surimi gel was weighed as W_1_ and centrifuged (8000 rpm, 15 min) at 4 °C. After centrifugation, the surface moisture of the sample was removed. The shrimp surimi gel after centrifugation was weighed as W_2_. The water-holding capacity was calculated according to the following equation:

*WHC (%)* = *W*_2_/*W*_1_ × 100

### 2.7. Low Field Nuclear Magnetic Resonance

The relaxation time tests of the shrimp surimi gel were conducted using a low field nuclear magnetic resonance (LF-NMR) analyzer (Niumag Electric Copporation, Shanghai, China). The CarrPurcellMeiboomGill (CPMG) was used to measure the relaxation times (T_2_) and peak area with test conditions as follows: the echo time (1000 ms), resonance frequency (20 MHz), number of scans (16), and echo counts (18,000).

### 2.8. SDS-PAGE

The samples (AK, Antarctic krill; AKS, Antarctic krill surimi gel; PWS, Pacific white shrimp; and PWSS, Pacific white shrimp surimi gel) were mixed with nine times volume SDS solution (5%, *w*/*v*) and stirred overnight to dissolve the total protein. The precipitation was removed by centrifugation (4000× *g*, 10 min) and the supernatant was retained. SDS-PAGE was performed at room temperature with 5% stacking gel and 10% separating gel. An amount of 40 μL protein solutions (2 mg/mL) were mixed with 10 μL loading buffer (2% sodium dodecyl sulfate, 12% glycerin, 5% 2-mercaptoethanol, 0.0025% bromophenol blue, and 0.0625 M Tris-HCl), and then heated at 100 °C for 5 min. The samples were centrifuged (10,000× *g*, 5 min) at 4 °C. Aliquots (15 μL) of the protein samples were loaded into the gel lanes. The gels were stained for 1 h with Coomassie Brilliant Blue-R250 and decolorized for 4 h in a decolorizing solution. The gray values were quantified by ImageJ software (ImageJ 1.47v National Institute of Health, Maryland, USA).

### 2.9. Cryo-Scanning Electron Microscopy (Cryo-SEM)

The shrimp surimi gels were cut into small pieces of 2 × 1 × 1 mm^3^ for microstructure observation. The gel pieces were set on a sample holder, and immersed in liquid Nitrogen. Then the sample holder was put into a cryogenic (−140 °C) preparation chamber under vacuum. Then, a blunt wedge was used to fracture the samples to expose the internal structure. All samples were sublimated (−65 °C, 30 min) and etched (60 s). Then, the shrimp surimi gels were observed at accelerating voltage (1.0 kV) with a SEM (SU8010, Hitachi Co., Tokyo, Japan).

### 2.10. Fourier-Transform Infrared (FTIR) Spectroscopy

The shrimp surimi gels were dried using a lyophilizer. Freeze-dried gel powder (2 mg) was ground with KBr (200 mg) and then pressed into a disk for further measurement. FTIR spectrum (4000–400 cm^−1^) of shrimp surimi gel was determined using a FTIR spectrometer (Spectrum Two, PerkinElmer Co., Japan) [16]. Air (25 °C) was recorded as the background spectrum. Protein secondary structure data were analyzed with OMNIC 9.2.

### 2.11. Molecular Forces

The shrimp surimi gels (3.0 g) were homogenized (10,000 rpm, 2 min) in five kinds of denaturing solutions (27 mL), including S1 (0.05 mol/L NaCl), S2 (0.6 mol/L NaCl), S3 (1.5 mol/L urea + 0.6 mol/L NaCl), S4 (8 mol/L urea + 0.6 mol/L NaCl), and S5 (0.5 mol/L β-Mercaptoethanol + 8 mol/L urea + 0.6 mol/L NaCl). Then the obtained liquid was stored (4 °C, 1 h) and centrifuged (15,000× *g*, 10 min). The concentrations of the dissolved protein were measured adopting the Bradford method, and differences in protein concentrations were used to determine the presence of molecular forces (ionic bonds, hydrogen bonds, hydrophobic interactions, and disulfide bonds) in protein gels [16].

### 2.12. Statistical Analysis

Data were analyzed from at least three independent experiments (*n* = 3) and expressed as mean ± standard deviation. The data were analyzed by SPSS 18.0 software (SPSS Inc., Chicago, IL, USA) and statistical significance of differences with *p* < 0.05 was evaluated with a Least significance difference (LSD) test.

## 3. Results and Discussion

### 3.1. Analysis of Gel Quality of Heat-Induced Antarctic Krill Surimi

**Figure 1:**
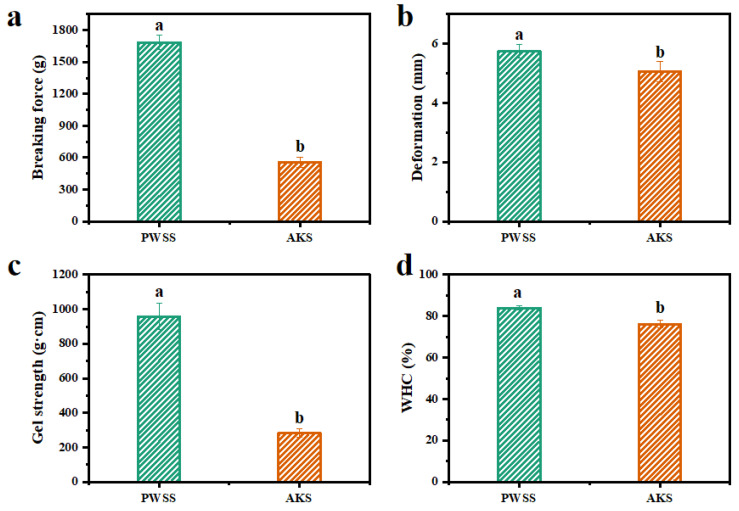
Gel strength and water-holding capacity of Antarctic krill and Pacific white shrimp surimi gels: (**a**) Breaking force, (**b**) deformation, (**c**) gel strength, and (**d**) water-holding capacity. Different letters indicate significant differences (*p* < 0.05). PWSS, Pacific white shrimp surimi; and AKS, Antarctic krill surimi.

The gel strength and water-holding capacity are important indexes for the evaluation of the quality of meat and meat products, because they are directly related to their acceptability to customers. Deformation and breaking force are related to the elasticity and gel strength of shrimp surimi gels, respectively [17,18]. Breaking force and deformation of the two kinds of shrimp surimi gels are shown in Figure 1a,b. The Antarctic krill surimi had lower breaking force compared with Pacific white shrimp surimi (*p* < 0.05) (Figure 1a), revealing that the interchain force maintaining the structural stability of Antarctic krill surimi was not as strong as that of Pacific white shrimp surimi. This might result in a low level of cross-linkage of myofibrillar proteins, which weakened the gel matrix of Antarctic krill surimi [19]. This was demonstrated by the result that the gel strength of Antarctic krill surimi was markedly lower than that of Pacific white shrimp surimi (*p* < 0.05) (Figure 1c). Pacific white shrimp surimi had high gel strength and dense microstructure [11]. As shown in Figure 1b, the deformation of Antarctic krill surimi gel was markedly weaker than that of Pacific white shrimp surimi gel (*p* < 0.05), reflecting that Antarctic krill surimi formed a low elastic gel. Therefore, both the gel strength and elasticity of Antarctic krill surimi were lower than those of Pacific white shrimp surimi. The protease of Antarctic krill has the characteristics of high activity and strong adaptability to low temperature, so the Antarctic krill will be rapidly autolyzed under the action of protease after death [6]. Earlier, Grantham reported that the autolysis of Antarctic krill weakened the protein gelation properties [6]. However, there was no report on the viscoelastic change trend of Antarctic krill surimi during heat-induced process.

Water-holding capacity is often used to assess the water retention capacity of shrimp surimi gels [20]. As shown in Figure 1d, the water-holding capacity of Antarctic krill surimi was markedly lower than that of Pacific white shrimp surimi (76.21% vs. 83.95%), indicating that the myofibril network interlacing ability of Antarctic krill surimi was weaker and thus less water could be retained or bound in the gel network [21]. A weak water retention capacity was not conducive to the formation of gel texture properties, which was consistent with the result of breaking force (Figure 1a). The lower water-holding capacity of Antarctic krill surimi might be due to the water having migrated out of the gel network during processing, indicating that the protein network structure of Antarctic krill surimi might be less compact than that of Pacific white shrimp surimi.

### 3.2. Changes in the Rheological Properties of Antarctic Krill Surimi during the Heat-Induced Gelation Process

Thermodynamic viscoelastic properties are closely associated with meat product development. As it is well known, storage modulus (G’) symbolizes solid-like behavior, whereas loss modulus (G’’) represents liquid-like behavior. Strain sweep tests were performed to measure the linear viscoelastic region. The linear viscoelastic region of both Antarctic krill surimi and Pacific white shrimp surimi was 0.1–10%. Therefore, the strain condition of Pacific white shrimp surimi and Antarctic krill surimi was set at 1%.

**Figure 2:**
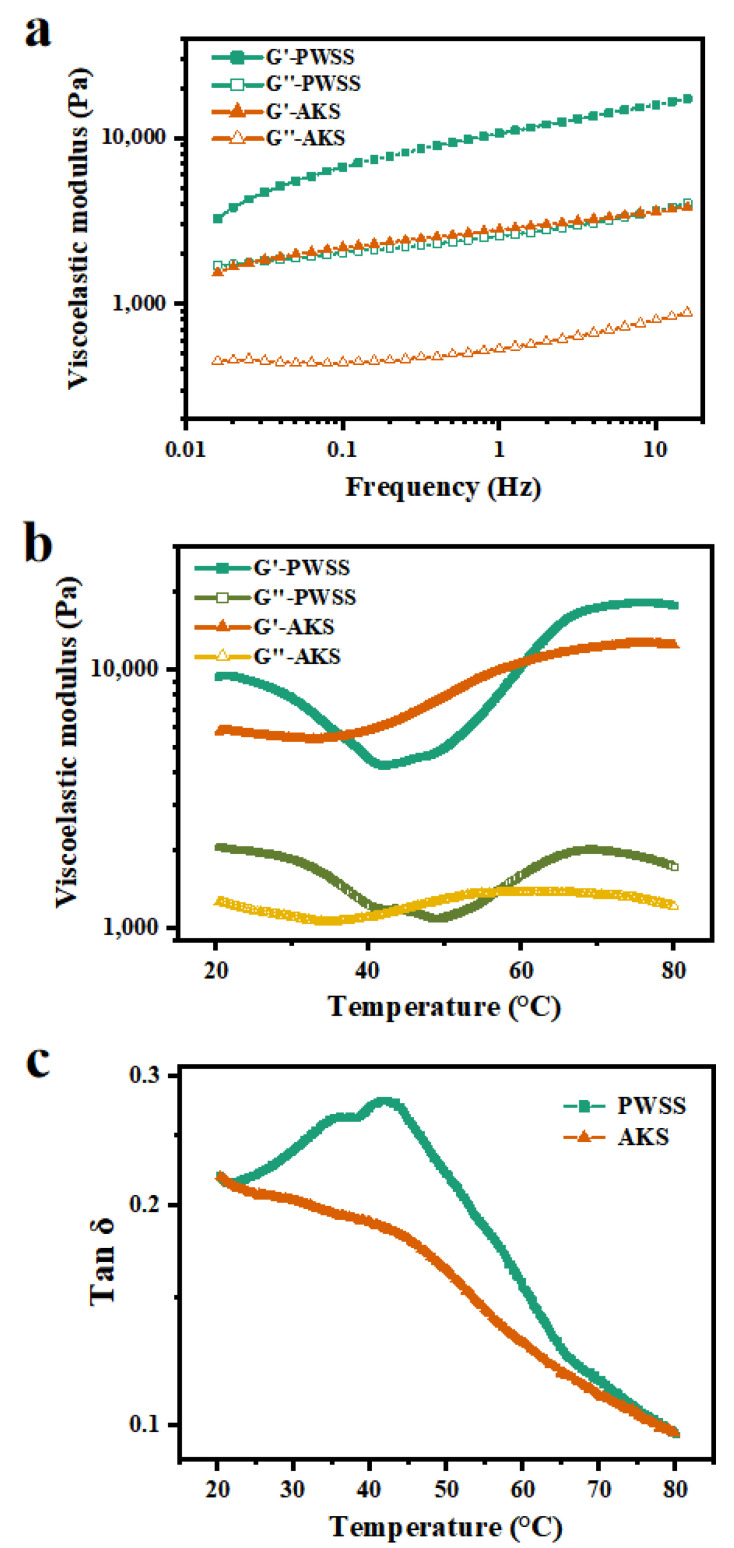
Viscoelastic properties of Antarctic krill and Pacific white shrimp surimi during the heat-induced gelation process: (**a**) Frequency sweep, (**b**) temperature sweep, and (**c**) tan δ. PWSS, Pacific white shrimp surimi; and AKS, Antarctic krill surimi.

The frequency sweep mainly reflected the relationship between mechanical properties and frequency of the material, and the variation of G’ and G’’ values with frequency is shown in Figure 2a. The two kinds of shrimp surimi exhibited high elasticity over the entire frequency range, with the G’ value consistently higher than the G’’ value, indicating that gelation was present in both cases. The G’ value of Antarctic krill surimi gel was lower than that of Pacific white shrimp surimi gel, revealing that the gel structure of Antarctic krill surimi was not as dense as that of Pacific white shrimp surimi, and the molecular chain flow was easier. The entire viscoelastic modulus increased with increasing frequency. The slope of the modulus of Antarctic krill surimi gel was smaller than that of Pacific white shrimp surimi gel, indicating the dependence of modulus on frequency [22]. Therefore, the elasticity and structural stability of Antarctic krill surimi gel were weaker than those of Pacific white shrimp surimi gel. This finding was consistent with the results of the texture analysis. 

Changes in G′ and G” of the two kinds of shrimp surimi during transition from sol to gel are shown in Figure 2b. With the change of temperature, the G ‘value of Antarctic krill surimi was always lower than that of Pacific white shrimp surimi. Generally, G’ is closely related to changes of sample elasticity during the gelling process [23]. The two kinds of shrimp surimi had higher G’ values at the initial stage (20 °C), which demonstrated the formation of a protein gel network via hydrogen bonds [24]. Thereafter, G ‘decreased with the increase of temperature and reached its lowest value around 40 °C. This phenomenon could be attributed to the degradation of the myofibrillar protein network mediated by endogenous proteolytic enzymes, resulting in enhanced protein mobility [25]. However, the variation of the G’ curve with temperature of Antarctic krill surimi was smaller than that of Pacific white shrimp surimi, which might be due to the enzymatic hydrolysis of Antarctic krill proteins after harvesting. G’ gradually increased with increasing temperature, probably because heat treatment promoted the interaction between myofibrillar proteins, thereby forming a stable gel network structure [26]. As reported by Buamard and Benjakul, unfolded proteins might facilitate intermolecular aggregation through domains or reactive groups [25]. Among them, the hydrophobic domains underwent cross-linking via hydrophobic interactions, while sulfhydryl groups were oxidized to form disulfide bonds [25]. Tan δ (G ‘/G’) was used to monitor the rheological properties of the gel during the heating process. Tan δ of a perfectly elastic substance is zero, and tan δ of a perfectly viscous substance is infinite. As shown in Figure 2c, the curve of tan δ showed the main type (the elasticity/viscosity dominant) of the formed shrimp surimi gel. Throughout the process of heat-induced gelation, the tan δ was <1, indicating that the elasticity of the sample plays a major role. During temperature ramping from 25 °C to 70 °C, the tan δ of Antarctic krill samples was always lower than that of Pacific white shrimp samples, indicating that the viscosity and cohesiveness of Antarctic krill samples were not as strong as those of Pacific white shrimp samples, which were stronger within the temperature range. 

### 3.3. Analysis of Water State and Distribution of Antarctic Krill Surimi after Heat-Induced Gel Formation

**Figure 3:**
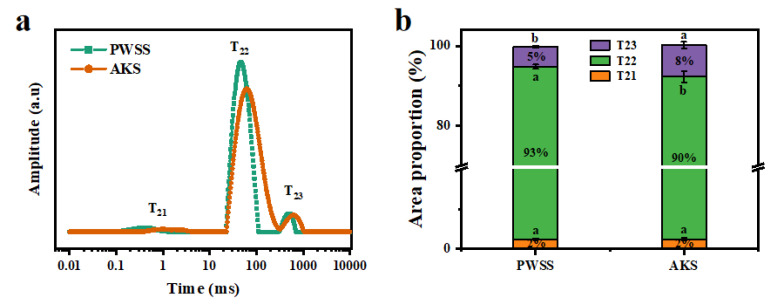
Nuclear magnetic resonance spin–spin relaxation (T_2_) of Antarctic krill and Pacific white shrimp surimi gels: (**a**) The curve of T_2_ relaxation time, and (**b**) the percentage of T_21_, T_22_, and T_23_. PWSS, Pacific white shrimp surimi; and AKS, Antarctic krill surimi. Different letters (a, b) within the same sample indicate significant differences (*p* < 0.05).

The relaxation time and peak area determined by LF-NMR are important indexes that illustrate the tightness between moisture and protein molecules. Three components (T_21_, T_22_, and T_23_) were observed in shrimp surimi samples (Figure 3a). The first peak (T_21_, bound water) corresponded to water molecules bound by strong hydrogen bonds. The second peak (T_22_) represented immobilized water, which was strongly bonded to the monolayer water molecules. The third peak (T_23_, free water) reflected weakly bound water molecules [27]. The T_22_ was the main component of water present in shrimp surimi gel. The study showed that the shorter the T_2_ relaxation time, the more hydrogen protons are bound, and the water was less free. As shown in Figure 3a, it could be clearly observed that the relaxation times (T_21_, T_22_, and T_23_) of Antarctic krill surimi were all longer than those of Pacific white shrimp surimi, indicating that the tightness between Antarctic krill surimi protein and water was lower than that of Pacific white shrimp surimi. This indicated that Antarctic krill surimi gel has weaker binding force to water and higher freedom of water than that of Pacific white shrimp.

The moisture composition (T_21_, T_22_, and T_23_) and content (A_21_, A_22_, and A_23_) of the two kinds of shrimp surimi are shown in Figure 3b. The immobilized water content (A_22_) of Antarctic krill surimi was lower than that of Pacific white shrimp surimi, but the free water content (A_23_) was higher than that of Pacific white shrimp surimi (*p* < 0.05). The results of gel strength and water distribution indicated that the three-dimensional network structure of Antarctic krill surimi gel might be weaker than that of Pacific white shrimp [28].

### 3.4. Three-Dimensional Microstructure of Heat-Induced Antarctic Krill Surimi Gel

**Figure 4:**
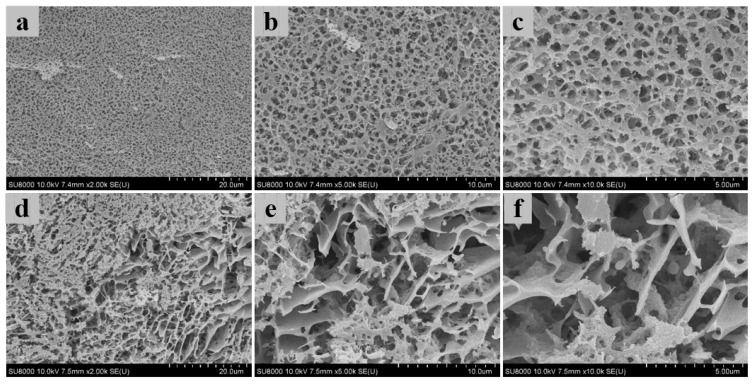
Cryo-SEM images of Antarctic krill and Pacific white shrimp surimi gels: (**a**) PWSS (2000×), (**b**) PWSS (5000×), (**c**) PWSS (10,000×), (**d**) AKS (2000×), (**e**) AKS (5000×), and (**f**) AKS (10,000×). PWSS, Pacific white shrimp surimi; and AKS, Antarctic krill surimi.

The microstructure of shrimp surimi gel is closely related to its texture properties and water distribution. The microstructures of heat-induced Antarctic krill surimi and Pacific white shrimp surimi gels were visualized by Cryo-SEM (Figure 4). The heat-induced Antarctic krill surimi gel had a rougher network with larger cavities or void, compared with Pacific white shrimp surimi gel. This was consistent with lower breaking force (Figure 1a) and water-holding capacity (Figure 1d). In contrast, a more ordered microstructure with higher interconnection was observed in Pacific white shrimp surimi gel. The results reconfirmed that the protein network of Pacific white shrimp surimi had a higher degree of cross-linking. The more ordered and dense network structure was more conducive to the water absorption of the gel [24]. This was consistent with the high water-holding capacity of Pacific white shrimp surimi gel and further verified the previous inferences. It has been reported that a rough microstructure might be related to the low unfolding degree of the α-helix [1].

### 3.5. Protein Patterns of Antarctic Krill Surimi with and without Heat Treatment

**Figure 5:**
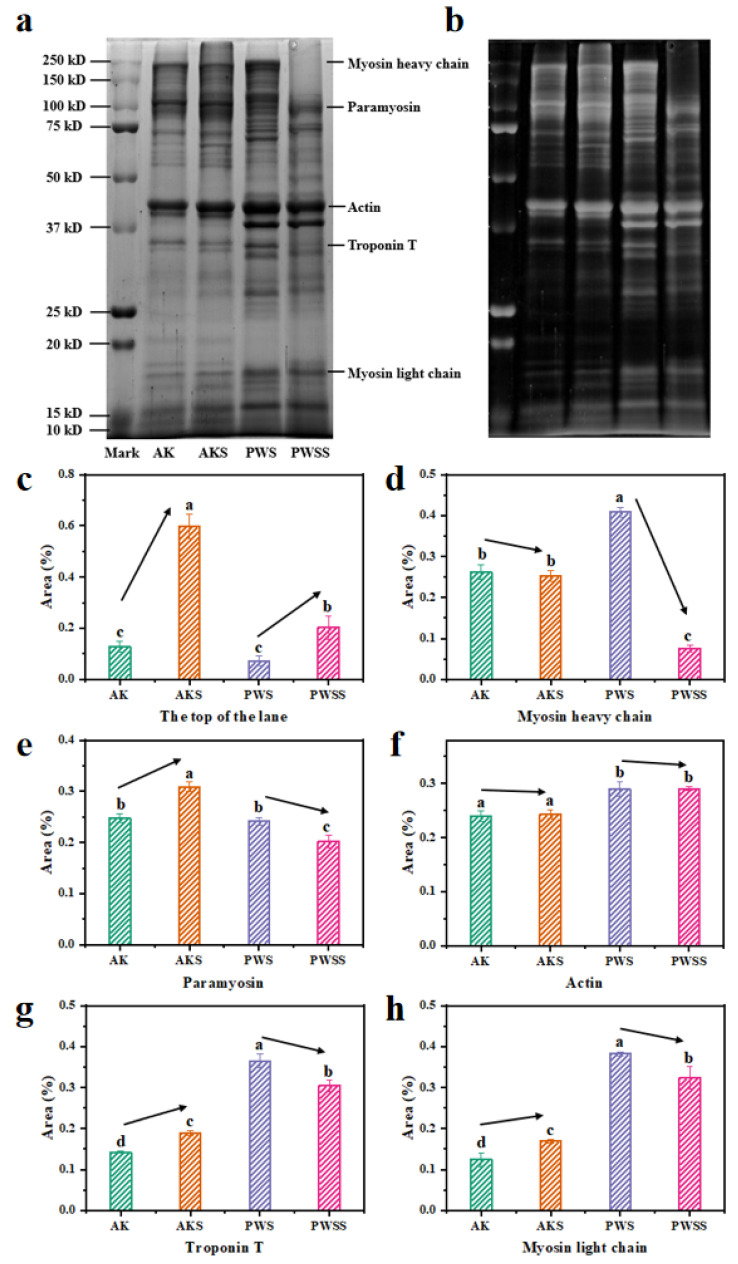
Protein patterns and gray value of Antarctic krill and Pacific white shrimp surimi (gels): (**a**) SDS-PAGE pattern, (**b**) grayscale map; (**c**) gray value of the top of the lane, (**d**) gray value of myosin heavy chain, (**e**) gray value of paramyosin, (**f**) gray value of actin, (**g**) gray value of troponin T, and (**h**) gray value of myosin light chain. AK, Antarctic krill; AKS, Antarctic krill surimi; PWS, Pacific white shrimp; and PWSS, Pacific white shrimp surimi. Different letters (a–d) indicate significant differences (*p* < 0.05).

Figure 5a,b depicts the SDS-PAGE patterns and Grayscale map of shrimp surimi gels with and without heat treatment, respectively. It can be seen that the two kinds of shrimp meats contained a wide variety of proteins, and the protein bands of Antarctic krill in the high molecular weight region (60–250 kDa) are greater than those of Pacific white shrimp, but the low molecular weight bands (10–60 kDa) are fewer than those of Pacific white shrimp. Salt-soluble protein plays a major role in the gelling properties of shrimp surimi, including myosin light chain (17 kDa), troponin T (34 kDa), actin (45 kDa), paramyosin (100 kDa), and myosin heavy chain (200 kDa) [29]. 

In Antarctic krill surimi gels, the protein content at the top of the lane in the heated groups (AKS and PWSS) was markedly higher than that in the unheated groups (AK and PWS) (*p* < 0.05) (Figure 5c,d). As reported by Singh and Benjakul, high levels of cross-linkage of proteins played an important role in the disappearance of myosin heavy chain band [30]. Myosin heavy chain plays a major role in the formation of protein networks [31]. In addition, the protein concentrations of paramyosin, troponin T, and myosin light chain bands in Pacific white shrimp surimi also decreased significantly after heat treatment (Figure 5e–h), which might also be due to protein cross-linking and aggregation caused by thermal denaturation. In Antarctic krill surimi gels, protein concentrations of paramyosin, actin, troponin T, and myosin light chain bands in the heated group were all higher than those in the unheated group (*p* < 0.05) (Figure 5e–h). This might be attributed to the hydrolysis of endogenous enzymes during heat treatment, resulting in the degradation of high molecular weight protein to low molecular weight protein [3]. Therefore, the low aggregation degree of myofibrillar protein had a severe impact on the formation of the gel network of Antarctic krill surimi, resulting in a less dense gel structure of Antarctic krill surimi than that of Pacific white shrimp.

### 3.6. Analysis of Protein Secondary Structure in Antarctic Krill Surimi Gel

**Figure 6:**
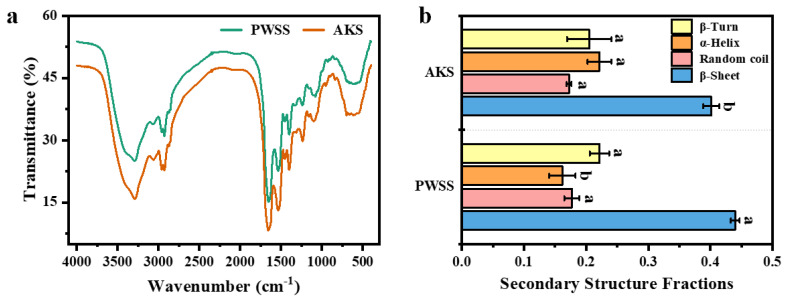
FTIR of Antarctic krill and Pacific white shrimp surimi gels: (**a**) FTIR spectra curve, and (**b**) the change of secondary structure. PWSS, Pacific white shrimp surimi; and AKS, Antarctic krill surimi. Different letters (a, b) within the same protein structure tested indicate significant differences (*p* < 0.05).

The protein secondary structure of Antarctic krill surimi gel has not been reported so far. FTIR is commonly used to study the changes of secondary structures in protein systems. Figure 6a shows the FTIR result of the shrimp surimi gels at 4000–400 cm^−1^. The trend of infrared curves of Antarctic krill surimi and Pacific white shrimp surimi were generally consistent, indicating that the chemical structures of Antarctic krill surimi gel were like that of Pacific white shrimp surimi gel. The amide A band (3500–3000 cm^−1^) is known as the “water region”, which is usually used to assess the interaction between protein and water. The groups (–OH and –NH) were helpful in forming the chemical bonds and were highly relevant to the strength of the hydrophobic interactions and hydrogen bonds [32]. The peak in Antarctic krill surimi gel at 3292.98 cm^−1^ shifted to a lower frequency of 3289.01 cm^−1^ in Pacific white shrimp surimi gel, which could be attributed to the complex vibrational stretches associated with free, inter-, and intramolecular hydroxyl groups [33]. Compared with the Pacific white shrimp surimi gel, Antarctic krill surimi gel had a higher peak frequency, which indicated that the intermolecular hydrogen bond of Antarctic krill surimi is not as strong as that of Pacific white shrimp surimi gel [34]. Studies have reported that the peak (3280 cm^−1^) might represent the presence of NH stretching vibrations, indicating multimolecular interactions, which might reflect the structural properties of paramyosin [11,35].

The amide I band (1700–1600 cm^−1^) is usually used to analyze the secondary structure of proteins (β-turn ~1680–1690 cm^−1^, β-sheet ~1670–1680 cm^−1^, random coil ~1660–1670 cm^−1^, and α-helix ~1645–1660 cm^−1^) [36]. As shown in Figure 6b, the β-sheet content of Antarctic krill surimi was significantly lower than that of Pacific white shrimp surimi (0.40 ± 0.01 vs. 0.44 ± 0.01), which meant that the gel structure of Antarctic krill surimi was not as ordered and stable as that of Pacific white shrimp surimi [37]. In addition, the α-helix content of Antarctic krill surimi was significantly higher than that of Pacific white shrimp surimi (0.22 ± 0.02 vs. 0.16 ± 0.02). This phenomenon is consistent with the result of SDS-PAGE. The above analysis indicates that β-sheet had a positive effect on gel properties, which partly explained the difference in gel formation between Antarctic krill surimi and Pacific white shrimp surimi.

### 3.7. Molecular Forces Involved in Antarctic Krill Surimi Gel Formation

The three-dimensional network skeleton of shrimp surimi gel is maintained by molecular forces between myofibrillar protein molecules (and their aggregates). Numerous analyses have shown that the myofibrillar protein structure changes during heat-induced process, which leads to altered interactions between protein molecules. As mentioned above, molecular forces (hydrogen bonds, ionic bonds, disulfide bonds, and hydrophobic interactions) are the main factors in maintaining the structure and quality of shrimp surimi gels [38]. Therefore, different force-disruption agents were used to determine the alteration of molecular forces involved in the formation of shrimp surimi gels.

As depicted in Table 1, the molecular forces of Antarctic krill surimi were all lower than those of Pacific white shrimp surimi (*p* < 0.05), which might be due to the low unfolding degree of Antarctic krill protein, resulting in less exposed active sites. This was consistent with the results of gel strength, SDS-PAGE, and SEM. Moreover, it could be found that disulfide bonds and hydrophobic interactions are higher than other molecular forces, suggesting that disulfide bonds and hydrophobic interactions played major roles in maintaining the gel properties of shrimp surimi. Heat treatment could induce the unfolding of the protein helix structure, exposing sulfhydryl groups and hydrophobic amino acids, and resulting in intermolecular interactions via disulfide bonds and hydrophobic forces [39]. Disulfide bonds played a major role in maintaining gel matrix stability [40]. In contrast, weak molecular forces might be one of the reasons for the poor gelation of Antarctic krill surimi. Therefore, the outcome of molecular forces could partly explain heat-induced gel differentiation between Antarctic krill surimi and Pacific white shrimp surimi.

### 3.8. Correlation Analysis and Principal Component Analysis

**Figure 7:**
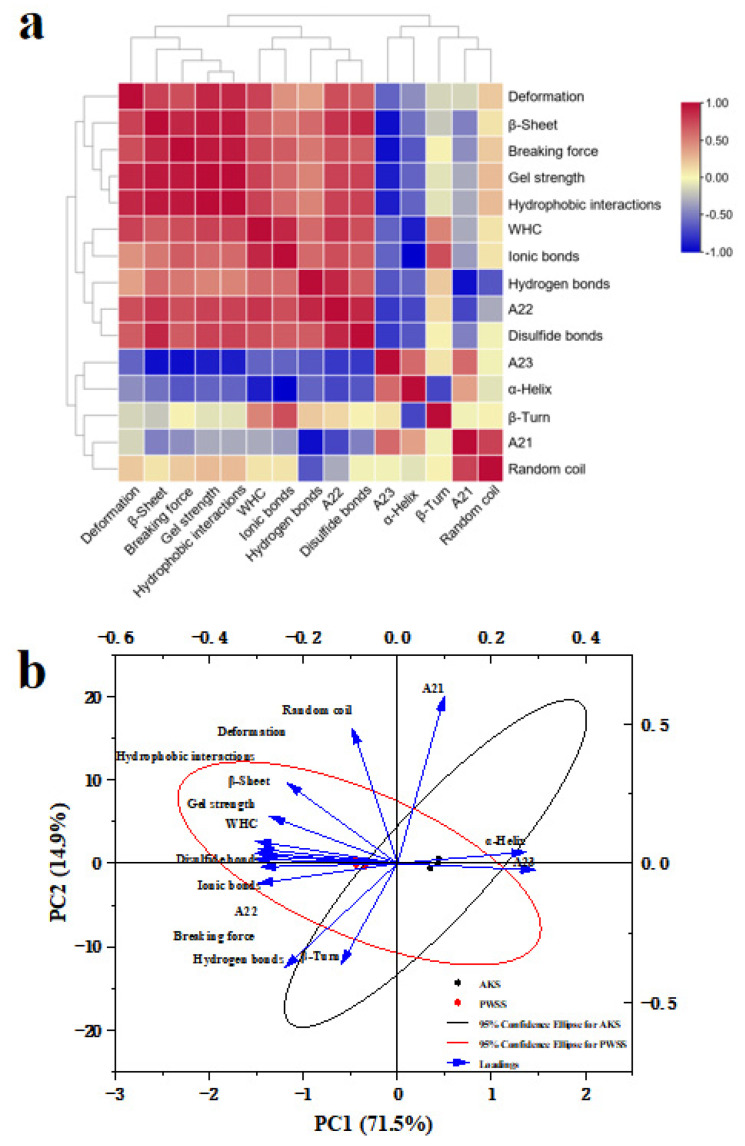
The correlation analysis and principal component analysis (PCA): (**a**) Correlation analysis; (**b**) the score scatter and loading plot of PCA. PWSS, Pacific white shrimp surimi; and AKS, Antarctic krill surimi.

Correlation analysis and PCA were conducted to clarify the relationship between the gel qualities (gel strength, breaking force, and deformation), the water properties (water-holding capacity, A21, A22, and A23), the protein secondary structure (β-turn, β-sheet, random coil, and α-helix) and the molecular forces (hydrogen bonds, ionic bonds, disulfide bonds, and hydrophobic interactions) of the two kinds of shrimp surimi. As shown in Figure 7a, water-holding capacity was positively related to the gel qualities (gel strength, breaking force, and deformation) of shrimp surimi, indicating that a strong water retention capacity was conducive to the formation of gel texture properties. The immobilized water (A_22_) in the myofibrillar protein lattice was positively correlated with the gel qualities, which indicated that the dense protein network structure contributed to the improvement of gel quality. Meanwhile, a positive correlation between molecular force and gel quality could be observed, and hydrophobic interaction was significantly positively correlated with the gel qualities of shrimp surimi, indicating that hydrophobic interaction played a major role in maintaining the stability of shrimp surimi. Moreover, there was a significant positive correlation between β-sheet and gel quality, indicating that β-sheet had a positive effect on gel properties. However, A21, A23, and α-helix were all negatively correlated with gel quality, and there was a significant negative correlation between free water (A23) and gel quality. The high content of free water indicated that the gel structure was loose, and the water retention capacity was relatively weak.

Through principal component analysis of each index of shrimp surimi, the contribution rate of PC1 and PC2 were 71.5% and 14.9%, respectively, and the cumulative contribution rate was 86.4% (>85%). The higher contribution rate could better reflect the information of the original index [41,42]. As shown in Figure 7b, the gel properties of Antarctic krill surimi were worse than those of Pacific white shrimp surimi. In contrast, Antarctic krill surimi had weaker water retention capacity and molecular forces, but higher α-helix and free water levels. Thus, the high α-helix / β-sheet ratio, weak intermolecular interactions, and low level of protein network cross-linkage were the main reasons for the poor quality of Antarctic krill surimi gels.

### 3.9. Feasibility Exploration of Polysaccharide Addition to Improve the Texture Properties of Heat-Induced Antarctic Krill Surimi Gel

**Figure 8:**
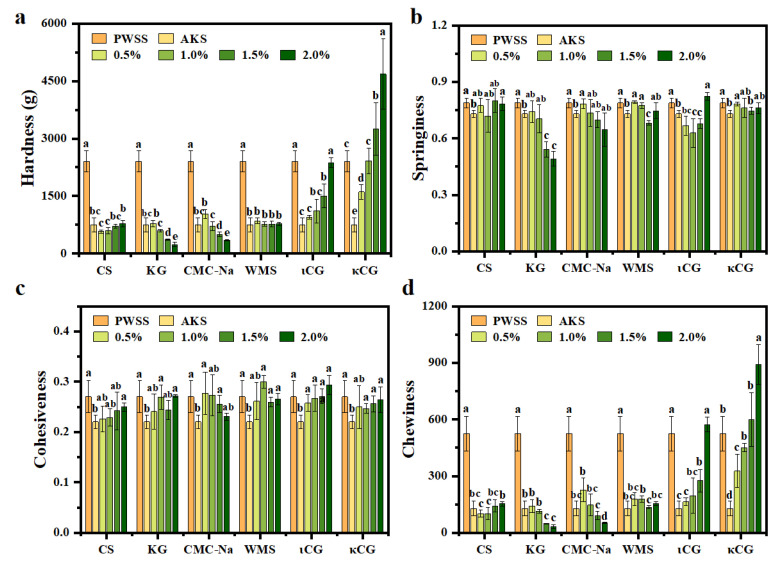
Texture properties of shrimp surimi gels: (**a**) Hardness, (**b**) springiness, (**c**) cohesiveness, and (**d**) chewiness. PWSS, Pacific white shrimp surimi; and AKS, Antarctic krill surimi; CS, chitosan; KG, konjac glucomannan; CMC-Na, sodium carboxyl methyl cellulose; WMS, waxy maize starch; ιCG, ι-carrageenan; and κCG, κ-carrageenan. Different letters within the same polysaccharide used indicate significant differences (*p* < 0.05).

In general, the effect of polysaccharides on the texture properties of protein gels depends on their molecular structure, and polysaccharides with different molecular structures can either inhibit or enhance the gel properties. Texture Profile Analysis mode utilizes a probe to compress a shrimp surimi sample twice to simulate chewing, which is often used to objectively describe the sensory properties of shrimp surimi, such as hardness, springiness, cohesiveness, and chewiness. Textural profiles of shrimp surimi gels are shown in Figure 8. Compared with Pacific white shrimp surimi, Antarctic krill surimi gel had a significant decrease in hardness (Figure 8a). Hardness refers to the maximum force during the first compression process and is usually related to the rupture strength [43]. Therefore, Antarctic krill surimi gels were easily crushed, indicating that their internal structure was not as dense as that of Pacific white shrimp surimi gels. There were significant differences in the effects of six polysaccharides on gel quality of Antarctic krill surimi. Among them, chitosan, konjac glucomannan, sodium carboxyl methyl cellulose, and waxy maize starch resulted in no significant improvement to the gel quality of Antarctic krill surimi (*p* > 0.05). It has been reported that the addition of excessive chitosan to surimi diluted the concentration of myofibrillar proteins, leading to a decrease in breaking force of surimi [44]. However, the addition of ι-carrageenan or κ-carrageenan significantly improved the gel quality of Antarctic krill surimi (*p* < 0.05). When ι-carrageenan (2%) or κ-carrageenan (1~2%) was added, the hardness of Antarctic krill surimi was similar to, or higher than that of Pacific white shrimp surimi. In addition, Antarctic krill surimi showed lower springiness and cohesiveness than Pacific white shrimp surimi (*p* < 0.05) (Figure 8b,c), reflecting that the molecular interactions maintaining the structural stability of Antarctic krill surimi were weaker than those of Pacific white shrimp surimi. The addition of polysaccharides improves the springiness and cohesiveness of Antarctic krill surimi, and increases with the increase of polysaccharide content. These results indicated that the addition of polysaccharides enhanced the molecular interaction of Antarctic krill surimi, which was beneficial to maintaining the stability of the gel structure. Moreover, the chewiness of Antarctic krill surimi was markedly lower than that of Pacific white shrimp surimi (128.06 ± 38.12 vs. 524.36 ± 91.78) (Figure 8d). This might be due to the denser tissue structure of Pacific white shrimp surimi, resulting in the tighter meat quality of Pacific white shrimp surimi. Thus, Antarctic krill surimi had worse mouthfeel than Pacific white shrimp surimi. By contrast, carrageenan could significantly enhance the chewiness of Antarctic krill surimi (*p* < 0.05), suggesting that the composite Antarctic krill surimi with ι-carrageenan (2%) or κ-carrageenan (1~2%) had better mouthfeel than Pacific white shrimp surimi. Therefore, the addition of ι-carrageenan (2%) or κ-carrageenan (1~2%) is an effective way to improve the gel quality of Antarctic krill surimi, and will contribute to the development of reconstituted Antarctic krill surimi products. The internal mechanisms by which carrageenan improves the gel properties of Antarctic krill surimi will be studied in depth in the future.

## 4. Conclusions

The results revealed the gel quality and gelation properties of heat-induced Antarctic krill surimi gel, and the possibility of improving the texture properties of Antarctic krill surimi by adding polysaccharides. The low aggregation degree of myofibrillar protein had a severe impact on the formation of the gel network of Antarctic krill surimi. As a result, breaking force, gel strength, water retention capacity, and viscoelasticity of Antarctic krill surimi were significantly lower than those of Pacific white shrimp surimi. Moreover, β-sheet structure and intermolecular forces had positive effects on the texture characteristics and gel matrix formation of shrimp surimi. Thus, the high α-helix / β-sheet ratio, weak intermolecular interactions, and low level of protein network cross-linkage were the main reasons for the poor quality of Antarctic krill surimi gels. On this basis, the effects of polysaccharides that could provide additional bonding to improve the texture properties of heat-induced Antarctic krill surimi gel were studied. It was found that the addition of ι-carrageenan (2%) or κ-carrageenan (1~2%) is an effective way to improve the gel quality of Antarctic krill surimi. These findings revealed the gelation properties of heat-induced Antarctic krill surimi gel, and the possibility of improving the texture properties of Antarctic krill surimi by adding polysaccharides, which would provide a point of reference for the in-depth development of Antarctic krill surimi with high nutritional quality.

## Figures and Tables

**Table 1:** Molecular forces involved in Antarctic krill and Pacific white shrimp surimi gels.

Samples	Molecular Forces in the Gel (mg/L)			
	Ionic Bonds	Hydrogen Bonds	Hydrophobic Interactions	Disulfide Bonds
PWSS	47.87 ± 2.45 ^a^	150.29 ± 1.18 ^a^	667.29 ± 17.16 ^a^	1677.41 ± 29.26 ^a^
AKS	39.66 ± 1.33 ^b^	144.69 ± 4.17 ^b^	536.17 ± 9.15 ^b^	1496.92 ± 23.75 ^b^

Different letters in a column indicate significant differences (*p* < 0.05).

## Data Availability

The data supporting the results of this study are included in the present article.
